# Ketogenic diet for status epilepticus in adult intensive care unit patients: a standard operating procedure

**DOI:** 10.1186/s42466-025-00431-x

**Published:** 2025-11-12

**Authors:** Katharina Feil, Daniela Schweikert, Michael Adolph, Sophia Kindzierski, Constanze Single, Felicitas Becker, Julian Bösel, Leona Möller, Annerose Mengel

**Affiliations:** 1 Department of Neurology & Stroke, University of Tübingen, Tübingen, Germany; 2 Hertie-Institute for Clinical Brain Research, University of Tübingen, Tübingen, Germany; 3 Nutrition Support Team, University Medicine Tübingen, Tübingen, Germany; 4 Department of Anesthesiology and Intensive Care Medicine, University Hospital Tübingen, Tübingen, Germany; 5 Department of Neurology, University of Ulm, Ulm, Germany; 6 Department of Neurology, University Hospital Heidelberg, Heidelberg, Germany; 7 Department of Neurology and Neurocritical Care, Johns Hopkins University Hospital, Baltimore, MD USA; 8 Department of Neurology, Friedrich-Ebert-Krankenhaus, Neumünster, Germany; 9 Department of Neurology, University of Marburg, Marburg, Germany

**Keywords:** Refractory status epilepticus, Supra-refractory status epilepticus, Ketogenic diet, Standard operational procedure

## Abstract

**Introduction:**

Refractory and super-refractory status epilepticus (RSE and SRSE) are life-threatening neurological emergencies with significant morbidity and mortality. When conventional treatment strategies, including antiseizure medications (ASM) and anesthetic agents, fail, alternative metabolic approaches, such as the ketogenic diet (KD), have been considered. KD is a high-fat, low-carbohydrate, and adequate-protein diet that induces ketosis, exerting anticonvulsant effects. Different types of KD are available, including the classical KD and the modified Atkins diet (MAD). Most published evidence in RSE and SRSE stems from classical KD protocols with high fat-to-carbohydrate/protein ratios (e.g., 4:1), whereas evidence for MAD in this setting is limited, and outcome equivalence across KD types remains unclear. While emerging evidence suggests efficacy in RSE and SRSE, clinical application in adult intensive care unit (ICU) settings remains inconsistent. This standard operating procedure (SOP), developed collaboratively by neurologists, intensivists, anesthesiologists, and clinical nutrition experts, provides a structured framework for classical KD implementation in adult ICU patients with RSE and SRSE, covering indications, contraindications, initiation, monitoring, and safety considerations.

**Definition:**

KD is a metabolic therapy that induces ketosis, providing an alternative energy source for the brain while modulating neurotransmitter activity and neuronal excitability.

**First steps:**

KD should be considered in RSE persisting for > 72 h despite adequate antiseizure treatment or in SRSE early after ICU admission, preferably within 24 h. Before initiation, metabolic screening is required, including tests on liver and renal function, lipid profile, electrolyte balance, and glucose metabolism. Absolute contraindications, such as severe hepatic dysfunction, ileus, or severe hypertriglyceridemia, must be ruled out. Continuous EEG monitoring should be considered. The diet is typically initiated using a classical KD protocol with a 4:1 lipid-to-non-lipid ratio formula and stepwise fat increase, supplemented with medium-chain triglyceride (MCT) oil to enhance ketosis. Essential micronutrients, including carnitine, vitamins, and trace elements, should be supplemented routinely based on clinical indication to prevent deficiencies.

**Comments:**

KD initiation follows a stepwise protocol with enteral formulations. Strict biochemical monitoring is essential to ensure metabolic stability, focusing on glucose, ketone levels, lipid profiles, liver function, and acid-base balance. Adverse effects, including metabolic acidosis, hypoglycemia, and hyperlipidemia, require timely adjustments. Involvement of an experienced dietician for nutritional support, trained nursing staff, as well as close interdisciplinary coordination are critical for successful initiation and maintenance of KD and for minimizing complications, particularly in centers with limited KD experience due to lack of familiarity with ketosis-impeding factors such as carbohydrate-containing medications. The suggested flowchart provides a structured approach for decision-making, highlighting indications, monitoring strategies, and emergency discontinuation criteria.

**Conclusion:**

This SOP provides a standardized, interdisciplinary protocol for classical KD implementation in adult ICU patients with RSE and SRSE. Developed collaboratively it integrates current literature and institutional experience. Despite heterogeneous study results, we advocate for early KD initiation in all SRSE patients without contraindications, given its safety, reversibility, and rapid implementability. By enhancing reproducibility and clinical feasibility, this SOP offers a practical and transferable tool for neurocritical care. We encourage other centers to adapt and apply this approach to further validate its utility.

## Introduction

Refractory status epilepticus (RSE) and superrefractory status epilepticus (SRSE) are serious neurological emergencies requiring prolonged intensive care management. Approximately 20–40% of status epilepticus (SE) cases progress to RSE, defined as seizures persisting despite treatment with at least one first-line agent (e.g., benzodiazepines) and one second-line anti-seizure medication (ASM) such as levetiracetam, phenytoin, valproate or phenobarbital. These cases frequently require continuous intravenous anesthetic agents (e.g., midazolam, propofol, or even thiopental) to achieve seizure control, [1–3].

Super-refractory status epilepticus (SRSE) is defined as SE that continues or recurs for ≥ 24 h after the initiation of anesthetic therapy or upon weaning from anesthetics [4] SRSE is highly resistant to standard treatment and, associated with increased morbidity and mortality [5]. The incidence of SRSE is estimated at 0.7 per 100.000, accounting [6, 7] for 5–10% of all SE cases. The mortality rate of SRSE (36%) is approximately twice as high as that of RSE, underscoring its poor prognosis. In addition to its acute severity, SE often follows a recurrent course with cumulative burden, further aggravating morbidity and mortality [8]. Exploratory approaches with agents such as ketamine and stiripentol underline the lack of robust evidence for pharmacological alternatives [9, 10]. Consequently, the management of SRSE often requires alternative adjunctive treatments such as immunotherapy, hypothermia, ketogenic diet (KD) and/or epileptic surgery in selected adult patients with limited evidence [3, 5, 11].

The KD is a high-fat, low-carbohydrate, and adequate-protein dietary regimen that induces a state of ketosis characterized by elevated serum ketone bodies and a shift in cerebral energy metabolism toward ketone utilization. Different types of KD are available, including the classical KD and the modified Atkins diet (MAD). Most published evidence in SRSE stems from classical KD protocols with high fat-to-carbohydrate/protein ratios (e.g., 4:1), whereas evidence for MAD in this setting is even more limited, and outcome equivalence across KD treatment types remains unclear.

Generally, KD exerts multiple anticonvulsant effects, including modulation of excitatory and inhibitory neurotransmitters, alteration of brain metabolism toward ketone utilization, anti-inflammatory effects, and mitochondrial function enhancement [7, 12]. These mechanistic insights provide the theoretical basis for applying high-fat, low-carbohydrate nutritional strategies, often enhanced by medium-chain triglycerides (MCT), to support seizure control in drug-resistant epilepsy.

In pediatric patients, KD has been widely established for more than a decade as an effective treatment option for drug-resistant epilepsy and is increasingly used in SRSE, with studies suggesting favorable seizure control outcomes [13–15]. 

In adult patients, however, data regarding KD in SE remain sparse and consist mostly of case reports, small case series, and observational studies with considerable heterogeneity in timing, diet composition, and monitoring strategies [5–7, 15–20]. In a retrospective single-center study, Francis et al. (2019) reported that 91% of adults with RSE achieved ketosis within one day of KD initiation, with seizure resolution in 73% and only manageable metabolic adverse effects [17]. A recent meta-analysis by Cornwall et al. (2023) [21] systematically reviewed available data on different SRSE treatments and found no significant association between the use of KD and improved outcomes across heterogeneous cohorts. However, given the variability in timing, protocol structure, and patient selection in the included studies, the authors acknowledged that beneficial effects in specific subgroups cannot be excluded and highlighted the urgent need for standardized treatment approaches. A prospective multicenter study evaluated the feasibility, safety, and efficacy of classical 4:1 KD in adults with SRSE. Among 24 screened patients, 15 were enrolled and treated with classical KD via gastrostomy tube, achieving ketosis within a median of 2 days and seizure termination in 79% of patients. Side effects such as metabolic acidosis, hyperlipidemia, and hypoglycemia were observed, but KD was overall feasible and well tolerated [16]. Some studies relied on only urinary ketosis, which does not correlate well with seizure control [13, 22]. In pediatric SRSE, parenteral ketosis induction has been explored as an alternative when enteral classical KD is unfeasible [13, 18]. 

On the other hand, critically ill ICU patients present additional challenges that may hinder KD therapy, including gastrointestinal dysfunction due to a low level of consciousness and immobility; prolonged coma, increased infection susceptibility, malnutrition, metabolic disturbances, and complex interactions between KD and concomitant treatments including treatments for epileptic activity [14]. Hidden carbohydrates in medications, infusions, and care products are a major barrier to achieving therapeutic ketosis, often requiring meticulous review of all treatments, particularly in centers with limited KD experience due to lack of awareness of ketosis-impeding factors. Careful monitoring, supplementation, and gradual weaning are essential to maximize seizure control and minimize complications [6, 7]. In summary, many questions remain regarding the optimal management of SRSE patients using KD in ICU settings, and further studies are needed to establish standardized protocols and assess long-term outcomes. Notably, KD offers specific practical advantages in the ICU setting for many SE patients, particularly those with (S)RSE: **i**t can be delivered via nasogastric tube or percutaneous endoscopic gastrostomy (PEG), and allows rapid, often point-of-care, monitoring of metabolic parameters and ASM plasma levels. While individual centers have published KD protocols for ICU use [18], these vary significantly in terms of initiation strategy, ratio adjustments, monitoring parameters, and the role of dietary supplements. Importantly, no standardized SOP exists that addresses the complexities of adult ICU patients in a structured, reproducible and interdisciplinary manner.

To address this gap, we developed a stepwise SOP for classical KD implementation in adult ICU patients with RSE and SRSE, grounded in both the literature and clinical experience. This SOP was designed in close collaboration with an interdisciplinary team including intensive care specialists and clinical nutrition experts. It adapts the well-established pediatric stepwise KD introduction method to the intensive care context of adult patients – an approach that, to our knowledge, has not yet been published in a standardized ICU format.

Our goal is to share this structured, literature-informed SOP to facilitate broader implementation, improve patient safety, and support future studies evaluating the role of KD in neurocritical care. Our protocol addresses both the lack of standardized KD protocols and the heterogeneity in timing and implementation strategies highlighted in recent evidence syntheses, by offering a reproducible and evidence-informed framework for early KD use in selected adult ICU patients.

## Standardized implementation of KD in adult ICU patients with RSE and SRSE

### Indications

KD should be considered in adult ICU patients meeting the following criteria:

- RSE lasting > 72 h, despite treatment with at least three different ASM or diagnosis of SRSE.
- Persistent seizure activity, reflected clinically and/or by continuous EEG.
- Early initiation after ICU admission, preferably within 24 h in cases where SRSE is anticipated, to maximize potential benefits and minimize treatment delays.

This early initiation strategy is based on growing evidence that metabolic interventions may be more effective if implemented early in the disease course [6, 7, 13, 16]. International consensus recommendations for New-onset Refractory Status Epilepticus (NORSE)/ Febrile infection-related epilepsy syndrome (FIRES) also advocate for the early use of KD as part of an aggressive treatment approach, recommending initiation as soon as possible after diagnosis [23]. Our protocol translates this principle into the adult ICU setting and advocates for classical KD initiation within 24 h if SRSE is clinically anticipated. However, the decision to initiate KD remains individualized and should consider the patient’s clinical trajectory, seizure persistence, and expected disease progression.

### Absolute contraindications

The contraindications listed are adapted from the International Consensus Statement on KD therapies in epilepsy by Kossoff et al. (2018) [24], with modifications based on our institutional SOP for adult ICU patients. Where evidence is limited in adult (S)RSE setting, recommendations reflect our local practice and thresholds derived from clinical experience in neurocritical care.

KD is not possible in patients with the following conditions:

- Severe hepatic failure (AST/ALT > 5× upper limit, ammonia > 5× upper limit, bilirubin > 15 mg/dL).
- Hypertriglyceridemia > 400 mg/dL or cholesterol > 300 mg/dL.
- Acute pancreatitis due to the high-fat content of KD and the risk of exacerbating pancreatic inflammation.
- Ileus, short bowel syndrome, or malabsorption syndrome.
- Diabetes mellitus type 1, pancreoprivic diabetes.
- Pregnancy.

### Relative contraindications (Require careful consideration and monitoring)

KD should only be initiated under strict monitoring in patients with:

- Concomitant propofol infusion (due to lipid load).
- Severe electrolyte disturbances (sodium < 120 or > 160 mmol/L) – must be corrected before initiation.
- Unstable hemodynamics requiring high-dose vasopressors.
- Ongoing gastrointestinal intolerance (e.g., persistent diarrhea).
- Concomitant treatment with carbonic anhydrase inhibitors (Topiramate, Zonisamide) should be used with caution due to an increased risk of metabolic acidosis and nephrolithiasis under KD.
- Septic shock.

## Initiation of KD

### Pre-initiation workup

Prior to starting KD, the following laboratory parameters should be assessed:

- Liver function tests (AST, ALT, bilirubin, ammonia).
- Renal function (creatinine, urea).
- Lipid profile (triglycerides, cholesterol).
- Electrolytes (sodium, potassium, calcium, magnesium).
- Glucose and ketone baseline levels.
- Pancreatic enzymes (amylase, lipase).

A nutrition support team (NST) should be consulted if possible, or individual protein and energy requirements should be calculated based on patient weight and metabolic status. Additional glucose administration (e.g. carrier solution) should be ruled out. Continuous electroencephalography (EEG) monitoring should be considered before and during the first three days of the ketogenic diet to assess treatment response.

The selected laboratory tests and baseline assessments are derived from published adult KD protocols [16–18] and were refined in collaboration with intensive care and clinical nutrition specialists. This multidisciplinary alignment ensures safety and consistency in the ICU context.

### Stepwise introduction of KD

Enteral KD is introduced gradually using a 4:1 lipid-to-non-lipid ratio formula (commercially available 4:1 liquid ketogenic formula with fibre, *e.g. KetoCal*^®^*4:1 LQ Multi Fibre*,* Nutricia*,* product name*) with stepwise fat increase: Day 1 (70%) → Day 2 (80%) → Day 3 (90%), achieved through a stages mixture of ketogenic formula and a standard isocaloric enteral formula (standard 1.0 kcal/mL enteral feed, *e.g. Nutrison*^®^*1.0*,* product name*), administered continuously at 50 mL/h over 20 h/day (Table 1).

**Table 1:** Stepwise feeding schedule

Day	Formula Composition	Infusion Rate	Total Infusion Time	Total Calories	Commercially available emulsified MCT oil (Separate Bolus Doses)
Day 1	600 mL commercially available nutritionally complete 4:1 ketogenic formula with fibre + 400 mL standard isocaloric enteral formula (standard 1.0 kcal/mL feed)	50 mL/h	20 h/day	1500 kcal (70% fat)	60 mL/day commercially available emulsified MCT oil, emulsified MCT oil 3 × 20 mL bolus via feeding tube at 08:00 AM, 2:00 PM, 8:00 PM
Day 2	800 mL commercially available nutritionally complete 4:1 ketogenic formula with fibre + 200 mL standard isocaloric enteral formula (standard 1.0 kcal/mL feed)	50 mL/h	20 h/day	1500 kcal (80% fat)	100–150 mL/day (3× ~33–50 mL per dose)
Day 3	1000 mL commercially available nutritionally complete 4:1 ketogenic formula with fibre (pure ketogenic formula)	50 mL/h	20 h/day	1500 kcal (90% fat)	150–200 mL/day (3× ~50–66 mL per dose)

This stepwise increase in lipid content (from 70% to 90%) is adapted from established pediatric epilepsy protocols [24, 25] and was transferred into the adult ICU context for the first time in our institution. Compared to other adult SRSE protocols (e.g [18]), which often start with fixed caloric targets, our approach focuses on tolerability and metabolic adaptation in critically ill patients.

From day 4 onward, supplementation with a medium-chain triglyceride (MCT) oil emulsion (commercially available emulsified MCT oil, e.g. *Liquigen*^®^,* Nutricia*,* product name*) is initiated to enhance ketosis. This provides rapidly absorbed MCTs, increases energy intake without raising carbohydrate or protein load, and supports patients who struggle to reach ketosis on standard enteral formulas. Titration proceeds as follows: Day 4: 45 mL/day, Day 5 75 mL/day Day 6 and onwards up to 100 mL/day, typically administered as 3 boluses per day.

**Fig. 1:**
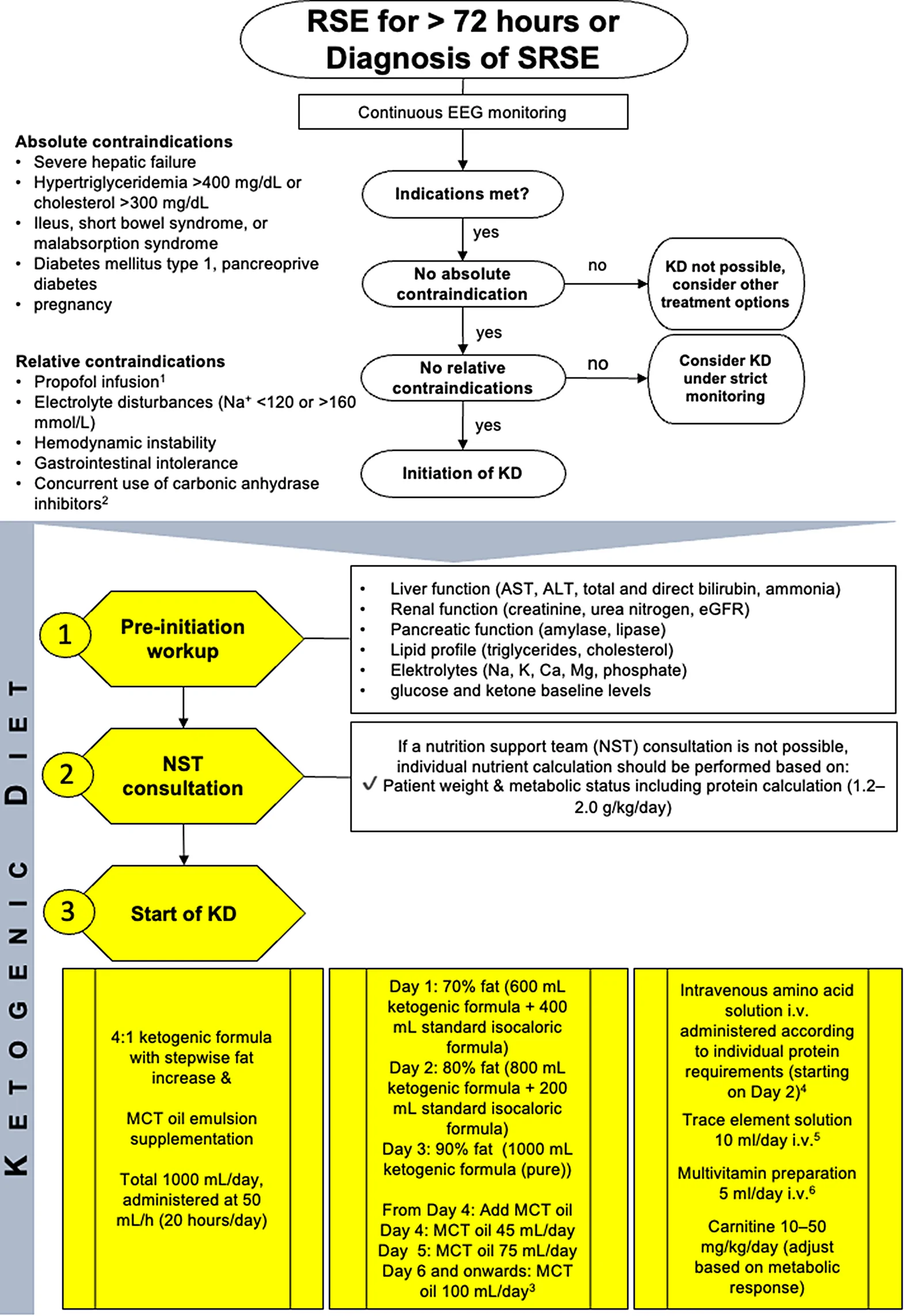

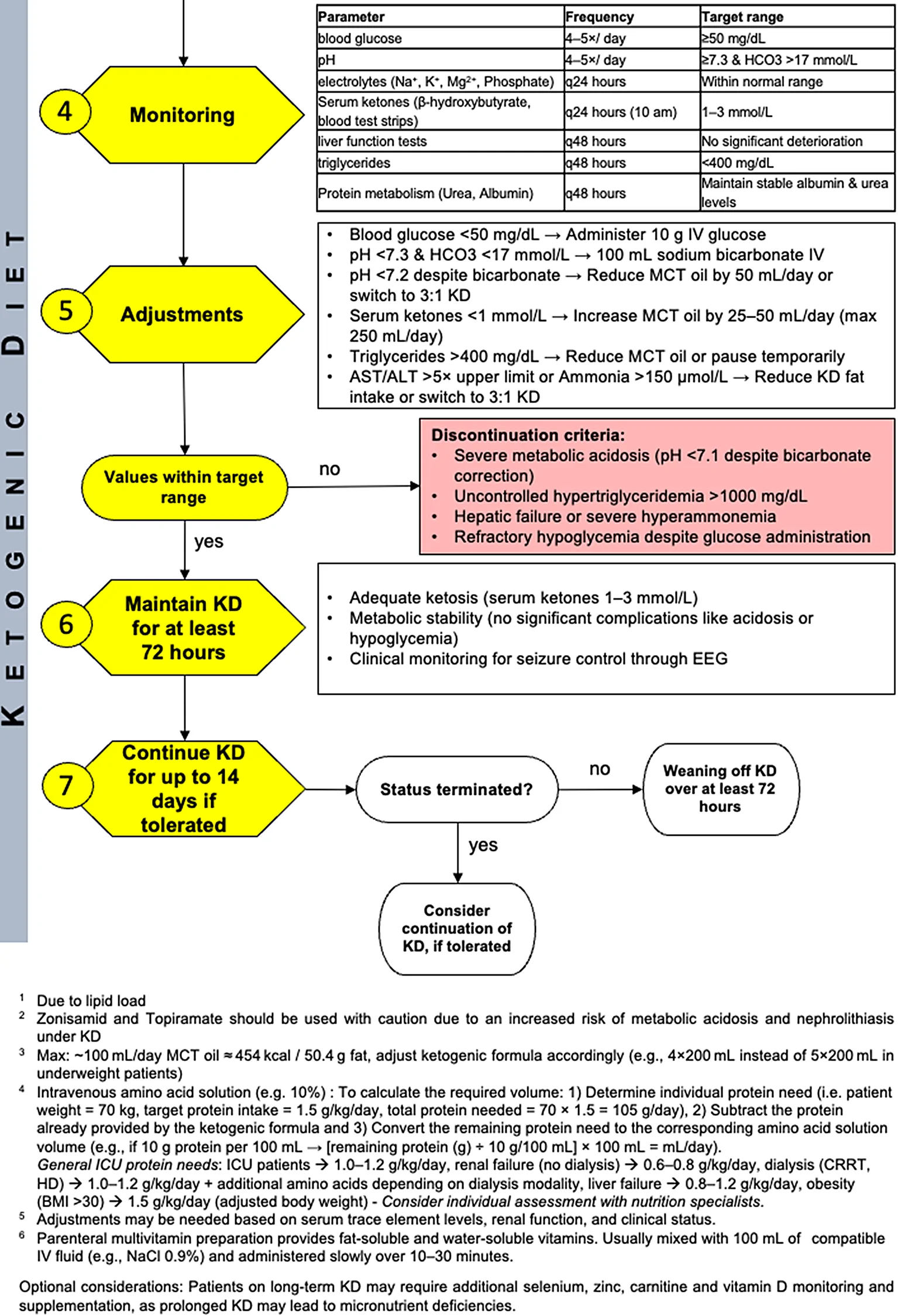
Flowchart of the standard operating procedure (SOP) for KD initiation, monitoring, and discontinuation in adult intensive care unit patients with RSE or SRSE. The algorithm outlines patient selection, contraindications, stepwise diet initiation, monitoring strategies, and emergency discontinuation criteria. Abbreviations: ALT, alanine aminotransferase; AST, aspartate aminotransferase; BMI, body mass index; Ca, calcium; CRRT, continuous renal replacement therapy; EEG, electroencephalogram; eGFR, estimated glomerular filtration rate; HCO₃, bicarbonate; HD, hemodialysis; ICU, intensive care unit; i.v., intravenous; KD, ketogenic diet; K, potassium; MCT, medium-chain triglyceride; Mg, magnesium; Na, sodium; NaCl, sodium chloride; NST, nutrition support team; RSE, refractory status epilepticus; SE, status epilepticus; SRSE, super-refractory status epilepticus; g/kg/day, grams per kilogram of body weight per day

Total MCT oil per day: Typically 50–200 mL/day, depending on patient needs and metabolic response and divided into multiple doses throughout the day, usually per enteral feed (Fig. 1).

MCT oil supplementation is supported by both pediatric and adult KD literature [16, 19] and was included in our SOP to enhance ketone availability, especially in cases with delayed ketosis or suboptimal metabolic response.

In the ICU setting, classical 4:1 KD remains the preferred formulation due to its metabolic efficiency, available standardized formulations, and superior evidence base for adult SRSE.

### Additional nutrition considerations (conditional, based on clinical indication)

- Parenteral protein supplementation: Consider continuous intravenous amino acid infusion (commercial 10% amino acid solution, e.g. *Aminoven*^*®*^*10% product name*) in patients with evidence of catabolism (e.g., persistently low albumin, elevated urea, documented muscle wasting) or high protein demand during prolonged ICU stays, aiming for protein intake of 1.0–1.2 g/kg bodyweight/day. Balance the potential risk of reduced ketosis from glucogenic amino acids against the risk of protein malnutrition.
- Micronutrient supplementation: Follow ICU nutrition guidelines, especially in patients with prolonged enteral/ parenteral nutrition or documented deficiencies:
  - Daily trace element/ micronutrient solution (commercial multi-trace element preparation, e.g. *Addel*^*®*^*, product name*), 10 mL in 200 mL sodium chloride over 1–2 h.
  - Daily infusion of a multivitamin preparation (commercial IV multivitamin formulation, e.g. *Cernevit*^*®*^*, product name*), 5 ml in 100 mL sodium chloride over 1–2 h)
- Carnitine Supplementation: Consider in patients with low plasma carnitine leverls of high risk for deficiency, particularly during prolonged KD and in those receiving valproate therapy, given the association with secondary carnitine depletion and impaired fatty acid β-oxidation [26]. Recommended dose: 10–50 mg/kg/day (adjusted based on metabolic response) day in the form of tablets or dissolved tablets.
- Protein Adjustments:
  - Increase amino acid dosage if signs of muscle breakdown (e.g. low albumin, high urea) persists for >48 h.
  - Reduce dosage in cases of renal dysfunction (e.g. elevated creatinine/urea).

## Monitoring, safety considerations and adjustments

Once KD is initiated, close metabolic, nutritional, and neurological monitoring is essential to ensure adequate ketosis, metabolic stability, and patient safety (see Table 2; Fig. 1). Serum ketones (β-hydroxybutyrate) should be measured every 8–12 h during the first 48 h, thereafter once daily. The target range is 1–3 mmol/L; an upper safety limit of > 5 mmol/L should prompt immediate action, including assessment of acid–base status, reduction of MCT or total fat intake, possible lowering of the KD ratio, and repeat ketone measurements every 4–6 h until values normalize. Continuous EEG monitoring should be considered to confirm SE and assess treatment response.

**Table 2:** Monitoring, safety considerations and adjustments

Parameter	Frequency	Target Range	Adjustments if value is out of range
Blood glucose	4–5× per day	≥ 50 mg/dL	If < 50 mg/dL, give 10 g IV glucose.
pH	4–5× per day	pH ≥ 7.3 & HCO3 > 17 mmol/L	If pH < 7.3 & HCO3 < 17 mmol/L, give 100 mL sodium bicarbonate IV If pH < 7.2 despite bicarbonate, reduce MCT oil by 50 mL/day and/or switch to 3:1 ketogenic formula
Electrolytes (Na, K, Mg, phosphate)	Every 24 h	Within normal range	If Na < 130 mmol/L: Hypertonic saline (NaCl 3%) 20–40 mL IV over 30 min. If K < 3.5 mmol/L: KCl IV 20–40 mmol/h if symptomatic. If Mg < 1.5 mg/dL: MgSO4 2 g IV over 1 h
Serum ketones (β-hydroxybutyrate, measured with blood test strips)	Every 8–12 h during the first 48 h after KD initiation, afterwards 1× daily (10.00 AM)	1–3 mmol/L	If ketosis is insufficient (< 1 mmol/L): increase MCT oil by 25–50 mL/day (max 250 mL/day) Serum ketones > 5 mmol/L → assess acid–base status, reduce MCT or fat intake, consider lowering the KD ratio, and monitor ketones every 4–6 h until levels fall below 5 mmol/L.
Liver function tests	Every 48 h	No significant deterioration	If AST/ALT > 5× upper limit, ammonia > 150 µmol/L, reduce KD fat intake or switch to 3:1 ketogenic formula
Triglycerides	Every 48 h	< 400 mg/dL	If triglycerides > 400 mg/dL or severe GI intolerance (persistent vomiting/diarrhea), reduce ketogenic formula infusion rate or temporarily pause MCT oil
Protein metabolism (Urea, Albumin)	Every 48 h	Maintain stable albumin & urea levels	If muscle breakdown (low albumin, high urea): increase amino acid infusion. If renal dysfunction (high creatinine/urea): reduce amino acid infusion.

### Emergency discontinuation criteria

If any of the following conditions persist despite appropriate corrective measures for more than 48 h, KD must be discontinued to prevent severe metabolic complications:

- Severe metabolic acidosis (pH < 7.1 despite bicarbonate correction).
- Uncontrolled hypertriglyceridemia > 1000 mg/dL.
- Hepatic failure or severe hyperammonemia.
- Refractory hypoglycemia despite glucose administration.

If KD discontinuation is required, a gradual transition to standard enteral nutrition is recommended to avoid rebound seizures and metabolic instability (see weaning strategy).

## Implementation barriers in the ICU

Successful implementation of KDT in the ICU requires an experienced multidisciplinary team, including a dietician familiar with ketogenic therapy, trained nursing staff, and pharmacists actively involved in reviewing all administered medications. Inadequate staffing or lack of KDT-specific expertise increases the risk of dietary errors and treatment failure. One of the most common pitfalls is the inadvertent administration of carbohydrates from non-nutritional sources, particularly in medications routinely used in the ICU. Hidden carbohydrate content has been documented in several benzodiazepine formulations (e.g., midazolam syrup), barbiturates (e.g., phenobarbital elixirs), and intravenous anti-seizure medications (e.g., valproate preparations containing propylene glycol), as well as in certain antibiotic formulations (e.g., clarithromycin suspensions), electrolyte solutions, oral care products, and nutritional supplements [6, 27]. These sources can substantially impair ketosis, prolong the time to achieve therapeutic ketone levels, and jeopardize seizure control. To minimize these risks, all medications, infusions, and care products should be systematically reviewed and substituted with carbohydrate-free formulations whenever possible before initiating KDT. A structured approach includes:

- Pharmacy-led review of all active and as-needed medications for carbohydrate content, with documentation in the medical record.
- Substitution with carbohydrate-free or low-carbohydrate alternatives where available (e.g., IV midazolam instead of oral syrup, sugar-free antibiotic formulations).
- Use of a standardized checklist (see Table 3) at therapy initiation and during daily rounds.
- Clear communication between ICU, pharmacy, and dietetics to ensure new orders are screened before administration.

Such systematic measures are essential to maintain ketosis and ensure safe and effective KDT delivery in the ICU setting.

**Table 3:** Checklist for implementation for KD in the ICU

Phase	Checklist item	Checked?
Before KD initiation	Multidisciplinary team (neurology, intensive care, dietetics, nursing, pharmacy) designated	
	Nutrition plan prepared and documented (classical KD, 4:1 ratio, stepwise initiation)	□
	Baseline labs obtained (liver function tests, lipid profile, electrolytes, glucose, ketones, amylase/lipase, carnitine in at-risk patients)	□
	Full review of all medications for carbohydrate content - carbohydrate-free alternatives prescribed where available □ Anti-seizure medications (e.g., midazolam syrup, phenobarbital elixir, valproate preparations containing propylene glycol) □ Antibiotics (e.g., clarithromycin suspension) □ Electrolyte solutions □ Infusion carrier solutions □ Care products (oral care, nutritional supplements)	□
	Monitoring plan established (EEG, labs, ketones, glucose, pH)	□
	Nursing staff briefed on KD-specific requirements	□
Daily KD checklist	Ketone levels within target range (1–3 mmol/L) / no exceedance > 5 mmol/L	□
	Blood glucose ≥ 50 mg/dL	□
	No new carbohydrate-containing medications prescribed	□
	No new glucose-containing infusion carriers administered	□
	MCT oil and KD formula administered as planned	□
	Supplementation as indicated (carnitine, vitamins, trace elements)	□
	No signs of complications (acidosis, hyperlipidemia, gastrointestinal intolerance)	□
	Interdisciplinary review (neurology, intensive care, dietetics, pharmacy) completed	□

## Duration and weaning strategy

KD should be maintained for at least 72 h before assessing the response. If effective and well-tolerated, it may be continued for up to 14 days, with ongoing assessment of seizure control and metabolic stability. If clinical improvement is observed but further therapy is needed, KD can be extended beyond 14 days under strict monitoring. However, if SE persists beyond 14 days under KD, KD should be gradually weaned off, and alternative treatment strategies considered.

Weaning off KD should happen over a minimum of 72 h to prevent rebound seizures. The transition should include:

- Gradual reintroduction of carbohydrates (e.g., standard enteral feed with increasing carbohydrate content).
- EEG monitoring during weaning.
- Close metabolic monitoring for hypoglycemia and acidosis.

## Comparison to other protocols

Several previously published KD protocols for SRSE (e.g [6, 7, 16, 18]). have addressed aspects of interdisciplinary monitoring and standardized thresholds for initiation and follow-up. Our SOP builds on these foundations by integrating these elements into a unified, stepwise framework adapted from pediatric protocols specifically for the adult ICU context. In particular, we combine early, graded initiation with predefined criteria for treatment initiation, escalation, and discontinuation, and operationalize these through an institution-wide, interdisciplinary implementation plan involving intensivists, neurologists, dietitians, and nursing staff. This approach aims to enhance feasibility, reproducibility, and safety while maintaining flexibility for individual patients’ needs.

## Conclusion

KD is a promising, safe and cost-effective adjunctive treatment of RSE and SRSE in adult ICU patients. This SOP provides a structured, interdisciplinary framework for the initiation, monitoring, and discontinuation of KD, ensuring metabolic safety while optimizing seizure control. It was developed collaboratively by neurologists, intensivists, anesthesiologists and clinical nutrition experts, with essential contributions from experienced ICU nursing staff, and is based on current literature and refined institutional experience with over 40 ICU-treated patients, integrating evidence with clinical feasibility. In addition to medical management, successful implementation critically depends on close collaboration with dieticians and trained nursing staff to avoid dietary errors, most importantly the inadvertent administration of carbohydrate-containing medications, which can impede ketosis. Interdisciplinary evaluation is essential for early recognition and management of complications or insufficient ketosis, particularly in centers with limited KD experience. Despite heterogeneous study results and inconclusive meta-analyses, we advocate for early KD initiation in all SRSE patients without contraindications, given its reversibility and rapid implementability when guided by a standardized protocol.

By offering detailed guidance on timing, nutritional strategy, monitoring parameters, and safety thresholds, this SOP promotes reproducibility and supports broader clinical adoption. In the absence of standardized ICU protocols and with increasing interest in adjunctive therapies, it serves as a practical and transferable tool for neurocritical care. We encourage other centers to adapt and apply this protocol to further validate its utility.

## Data Availability

Data sharing is not applicable to this article as no datasets were generated or analysed during the current study.

## Abbreviations

ALT: Alanine aminotransferase
ASM: Antiseizure medication
AST: Aspartate aminotransferase
BMI: Body mass index
Ca: Calcium
CRRT: Continuous renal replacement therapy
EEG: Electroencephalogram
eGFR: Estimated glomerular filtration rate
FIRES: Febrile infection-related epilepsysyndrome
HCO₃: Bicarbonate
HD: Hemodialysis
ICU: Intensive care unit
i.v.: Intravenous
KD: Ketogenic diet
K: Potassium
MCT: Medium-chain triglyceride
Mg: Magnesium
Na: Sodium
NaCl: Sodium chloride
NORSE: New-onset Refractory Status Epilepticus
NST: Nutrition support team
RSE: Refractory status epilepticus
SE: Status epilepticus
SRSE: Super-refractory status epilepticus
g/kg/day: Grams per kilogram of body weight per day
